# Robustness of Propofol and Sevoflurane on the Perioperative Immune Function of Patients Undergoing Laparoscopic Radical Nephrectomy

**DOI:** 10.1155/2022/1662007

**Published:** 2022-01-30

**Authors:** Jiming Wang, Shaomin Cui, Liang Kong, Bin Ma, Jianhua Gu

**Affiliations:** Department of Anesthesiology, Jinan People's Hospital Affiliated to Shandong First Medical University, Jinan 271199, Shandong, China

## Abstract

*Objective*. This study aimed to evaluate the robustness of propofol combined with sevoflurane in patients undergoing laparoscopic radical nephrectomy and its influence on immune function during perioperative period. A total of 140 patients who underwent laparoscopic nephrectomy in the Department of Oncology of our hospital from January 2018 to January 2020 were divided into the control group and the study group by the random number table method, with 70 cases in each group, who were given sevoflurane anesthesia and sevoflurane combined with propofol anesthesia, respectively. The anesthesia effect and perioperative immune function of the two groups of patients were compared. No remarkable difference was observed in the extubation time, breathing recovery time, and awakening time between the two groups; the extubation coughing score and postextubation restlessness score of the study group were significantly lower than those of the control group; the postoperative renal function indexes of the two groups of patients were not considerably different; after treatment, the CD3+ (%), CD4+ (%), and CD4+/CD8+ of the two groups of patients decreased significantly, with a higher level in the study group. For patients undergoing laparoscopic radical nephrectomy, the combination of propofol and sevoflurane yields a promising outcome in enhancing the anesthesia results and improving the perioperative immune function.

## 1. Introduction

Renal cell carcinoma, also known as renal cancer, is a malignant tumor originating from the urinary tubule epithelial system of the renal parenchyma, including various renal cell carcinoma subtypes coming of different parts of the urinary tubule [1, 2]. Surgery is the preferable choice for early- and middle-stage renal cancer patients, including nephron-sparing surgery and radical nephrectomy [3, 4]. Recently, studies have also proven that laparoscopic radical nephrectomy is equivalent to open radical nephrectomy in terms of tumor control. In this regard, the low-invasive laparoscopic radical nephrectomy has increasingly become acceptable in the field of urology [5, 6].

The immune system plays a key role in the occurrence, development, metastasis, and prognosis of tumors, and the impaired immune function caused by surgery would increase the risk of tumor recurrence or metastasis [7]. Studies have shown that intravenous anesthetics could exert a positive impact on the immune function of patients and thus in turn hinder the progression of malignant tumors [8]. Propofol, a short-acting intravenous anesthetic, is predominantly used for the induction and maintenance of general anesthesia and enjoys tremendous popularity in laparoscopic surgery [9]. Propofol, characterized by stable induction and speedy recovery from anesthesia, has been extensively used as an intravenous anesthetic in tumor removal surgery. Studies pointed out that in addition to anesthesia, propofol can protect the immune system function, thereby inhibiting the growth of tumor cells and improving the prognosis of patients. Its application has been frequently reported in malignant tumors such as breast cancer, glioma, and pancreatic cancer, yet there is a paucity of evidence on the impact of the immune function of patients undergoing radical nephrectomy [10]. Sevoflurane is an emerging inhaled anesthetic, characterized by low blood/gas distribution coefficient, no respiratory tract irritation, short postoperative recovery time, and excellent anesthesia efficiency, has a muscle relaxation effect in addition to sedation [11].

This study aims to explore propofol combined with sevoflurane anesthesia for patients undergoing laparoscopic radical nephrectomy. The results are reported as follows.

## 2. Materials and Methods

### 2.1. General Information

A total of 140 patients with ASA I-II who underwent laparoscopic nephrectomy in the oncology department of our hospital from January 2018 to January 2020 were selected and divided into the control group and the study group according to the random number table method and based on the ratio of 1 : 1, 70 cases in each group. All patients were informed of the study plan before enrollment, and informed consent was obtained from the patients and their families. The design of this study was approved by the ethics committee of our hospital with the approved no. of 2017–23/442, and all the procedures in the study conformed to the Declaration of Helsinki [12].

### 2.2. Inclusion and Exclusion Criteria

#### 2.2.1. Inclusion Criteria

(1) Renal cancer was diagnosed clinically and pathologically and scheduled to perform laparoscopic radical nephrectomy; (2) age ≥ 60 years old; (3) ASA grade II ∼ III; and (4) body mass index (BMI) was between 20 ∼ 28 kg/m^2^.

#### 2.2.2. Exclusion Criteria

(1) Distant metastasis of cancer cells and small renal cancer that can be partially performed nephrectomy; (2) patients with tumor invading renal vein, inferior vena cava, or venous tumor thrombus; (3) other previous kidney or abdominal surgery history; (4) allergy to anesthetics and other drugs in the study; (5) severe liver and kidney function or other organ dysfunction; and (6) inability to cooperate due to anxiety, depression, and other mental illnesses or organic mental disorders.

### 2.3. Anesthesia

All patients were fasted for 8 hours before operation, and the Philips M8001 A multifunction monitor was used to monitor the patients' blood pressure, heart rate, pulse, blood oxygen saturation, electrocardiogram, and other indicators.

All patients were treated with sufentanil (Sinopharm Group Industrial Co., Ltd., approval no. H20123297), etomidate (Jiangsu Hengrui Pharmaceutical Co., Ltd., approval no. H32022379), and cisatracurium bensulfonate (Shangyao Dong British Pharmaceutical Co., Ltd., approval no. H20123332) to induce anesthesia; the specific dosage is as follows: sufentanil 0.4–0.6 *μ*g/kg, etomidate 0.15–0.30 mg/kg, and cisatracurium benzalkonium 0.15–0.20 mg/kg were injected successively for induction of anesthesia; after the patient's consciousness lost, an enhanced tracheal intubation was inserted through the mouth to connect the anesthesia machine and the ventilator, with the tidal volume of 6–8 ml/kg, the respiratory rate of 12–18 times/min, and the positive end expiratory pressure of 3–5 cmH_2_O.

Sufentanil was used to maintain anesthesia in both groups of patients at a dose of 8–20 *µ*g/(kg·h); on this basis, the control group additionally used inhalation of 2%–4% sevoflurane (Shanghai Hengrui Pharmaceutical Co., Ltd., approval no. H20070172) to maintain anesthesia; the study group was additionally given propofol on the basis of the control group (Guangdong Jiabo Pharmaceutical Co., Ltd., approval no. H20051842) 4–12 mg/(kg·h) to maintain anesthesia.

All patients were guided by the bispectral index (BIS) during anesthesia, and the BIS value was controlled to be 40–60.

### 2.4. Major Outcomes

Renal function indexes: The fasting venous blood sample was drawn in the morning before induction of anesthesia (T0), 1 h after the beginning of operation (T1), completion of operation (T2), 1 day after operation (T3), and 3 days after operation (T4). The automatic biochemical analyzer (7150 Hitachi, Japan) was used to monitor serum creatinine (Scr) and blood urea nitrogen (BUN).

Immune function indicators: The fasting venous blood sample was drawn in the morning before and 7 days after the operation. Flow cytometry (BD FACS CALIBUR, USA) was used to analyze the percentages of CD3+ T cells, CD4+ T cells, and CD8+ T cells, and the ratio of CD4+/CD8+ cells was calculated. The phycoerythrin and fluorescein isothiocyanate two-color direct-labeled immunofluorescence kit and FSC/SSC gating were used to obtain 1 × 10^4^ cells.

### 2.5. Secondary Outcomes

Basic perioperative data: the patient's extubation time, breathing recovery time, wake-up time, extubation choking score, postextubation restlessness score, and other indicators were recorded to evaluate the effect of anesthesia.

Postoperative adverse reactions: All patients were followed up for 7 days after operation. The occurrence of adverse reactions such as hypotension, nausea and vomiting, and hypoalbuminemia were observed and recorded, and the incidence of adverse reactions was calculated.

### 2.6. Statistical Analysis

The SPSS 23.0 software was used for statistical analysis and the GraphPad prism 8.0 software for graphic plotting. The enumeration data were expressed as rate, and the chi-square test was used for the comparison; the measurement data were expressed as mean ± standard deviation (*x* ± *s*) and analyzed by the *t*-test and analysis of variance. The statistical difference was assumed at *α* = 0.05.

## 3. Results

### 3.1. General Information

As shown in Table 1, the differences in age, gender, BMI, ASA grade, TNM stage, tumor location, and tumor diameter between the two groups were not statistically significant (all *P* > 0.05).

### 3.2. Comparison of Extubation Time, Breathing Recovery Time, and Wake-Up Time

As shown in Table 2, there was no statistically significant difference in extubation time, breathing recovery time, and wake-up time between the two groups of patients (all *P* > 0.05).

### 3.3. Comparison of the Extubation-Induced Coughing Score and the Post-Extubation Restlessness Score

**Figure 1:**
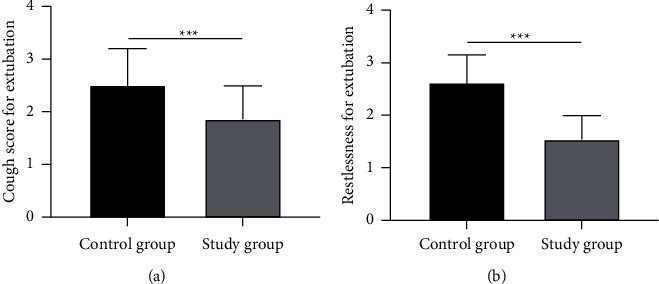
Cough score and restlessness score for extubation. (a) Cough score for extubation; (b) Restlessness score for extubation; ^*∗∗∗*^*P* < 0.001.

The extubation-induced coughing score and postextubation restlessness score of the study group were significantly lower than those of the control group (all *P* < 0.05, Figure 1).

### 3.4. Comparison of Renal Function Indexes at Different Time Points

**Figure 2:**
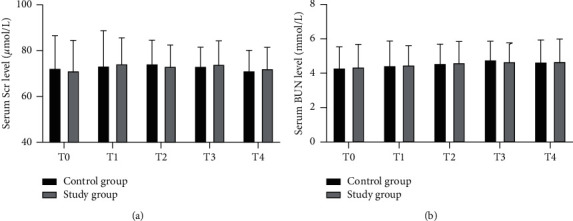
Serum levels of Scr and BUN at different time points. Scr, serum creatinine; BUN, blood urea nitrogen. (a) Serum levels of Scr and (b) Serum levels of Scr BUN.

As shown in Figure 2, no remarkable difference was observed in Scr and BUN levels between the two groups at different time points (all *P* > 0.05). Also, there was no statistically significant difference in the levels of Scr and BUN in the group at T1 to T4 as compared with T0 (all *P* > 0.05).

### 3.5. Comparison of Postoperative Pain

As shown in Table 3, the VAS score of the study group was considerably lower than that of the control group immediately after waking up and 12 hours after waking up (all *P* < 0.01); 24 hours after waking up, there was no significant difference in the VAS score between the two groups (*P* > 0.05).

### 3.6. Comparison of Perioperative Immune Function

**Figure 3:**
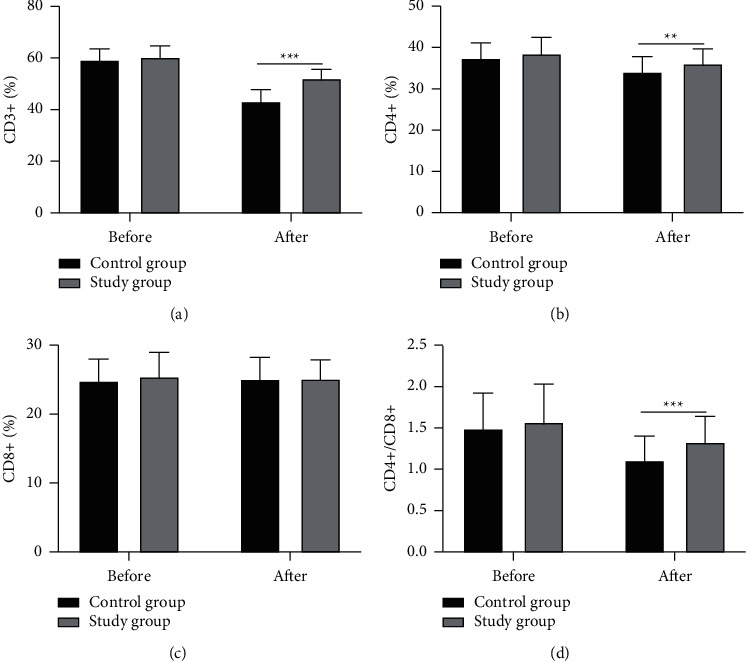
Comparison of perioperative immune function between the two groups. (a) CD3+(%), (b) CD4+(%), (c) CD8+(%), and (d) CD4+/CD8+(%). ^*∗∗*^*P* < 0.01; ^*∗∗∗*^*P* < 0.001.

As shown in Figure 3, the two groups witnessed no distinctive difference in the preoperative CD3+, CD4+, CD8+, and CD4+/CD8+ (all *P* > 0.05). After treatment, CD3+ (%), CD4+ (%), and CD4+/CD8+ in both groups decreased significantly, with better results observed in the study group (all *P* < 0.05), whereas CD8+ (%) demonstrated no evident change (*P* > 0.05).

### 3.7. Comparison of Adverse Reactions

As shown in Table 4, both groups had no hypotension after operation. In the control group, there were 2 cases of fever, 1 case of nausea and vomiting, 1 case of hemoglobin reduction, and 2 cases of other adverse reactions, with the total adverse reaction rate of 8.57% (6/70); in the study group, there was 1 case of fever, 3 cases of nausea and vomiting, 2 cases of hemoglobin reduction, and 2 cases of other adverse reactions, with the total adverse reaction rate of 11.43% (8/70). Overall, the two groups revealed no marked difference in postoperative adverse reactions (*P* > 0.05).

## 4. Discussion

In this study, the postoperative VAS score of the study group was significantly lower than that of the control group. Sevoflurane is a new halogen inhalation anesthetic. Compared with other inhalation anesthetics, sevoflurane has the advantages of rapid induction, small respiratory tract irritation, low solubility, rapid absorption and clearance, rapid recovery, easy adjustment of anesthesia depth, light inhibition of circulation, and certain muscle relaxation [13]. Propofol is a new type of rapid short-acting intravenous anesthetics, with rapid onset, stable induction, short duration, complete awakening, without causing nightmares and hallucinations, and other mental symptoms. Combined use of sevoflurane and propofol can significantly improve postoperative pain [14].

In this study, propofol combined with sevoflurane was used for anesthesia, and patients obtained more favorable immune function after surgery. Studies have confirmed that the choice of intraoperative anesthetics has a crucial role in postoperative immune function, and some inhaled and intravenous anesthetics inhibit cellular immunity, especially T lymphocytes [15]. T cells, the main components of lymphocytes, exert a variety of biological functions such as killing of target cells, assisting or inhibiting the production of antibodies by B cells, responding to specific antigens and mitogens, and producing cytokines. As a consequence, it plays a major part in suppressing the occurrence and development of tumor. CD3+ T cells represent total T cells, including CD4+ T cells and CD8+ T cells. [16]. Among them, CD4+ T cells, also known as helper T cells, function in assisting humoral immunity and cellular immunity; CD8+ T cells, also called cytotoxic T cells, function in killing target cells. CD3+, CD8+, and CD4+/CD8+ are common indicators to evaluate the immune function of the body [17]. In this study, the abovementioned indicators of the two groups of patients decreased after surgery, confirming the side profile of surgical operations on immune function. Additionally, the abovementioned indicators of the study group were significantly higher than those of the control group, suggesting that the combination of propofol significantly improves the immune function of the body. To our best knowledge, propofol, a commonly used anesthetic, is characterized by rapid awakening, quick onset, and short action time, thereby being widely used in the induction and maintenance of general anesthesia. In addition to anesthesia, it also has a certain antitumor effect. Studies have shown that propofol can regulate human immune function, for example by affecting the activity of immune cells and cytokines. Propofol promotes cytotoxic T cell activity in mice and humans. In a mouse model of breast cancer, propofol did not reduce NK cell activity and was not associated with lung cancer cell metastasis. Research on the effect of propofol on the immune function of cancer patients by Ren et al. showed that propofol significantly increases the expression of CD4+ T cells and effectively reduces the concentration of cortisol, confirming the role of propofol in improving immune function and reducing stress response [18]. Moreover, previous studies revealed that propofol inhibits the biological activity of cancer cells in intraoperative anesthesia for pancreatic cancer, gastric cancer, liver cancer, lymphoma, and cervical cancer [19–21].

## 5. Conclusion

In patients undergoing laparoscopic radical nephrectomy, the use of propofol combined with sevoflurane augments the anesthesia outcomes and boosts the perioperative immune function, yet its mechanism needs to be further explored.

## Figures and Tables

**Table 1:** Comparison of general information.

	Control group	Study group	*t*/*χ*2	*P* value
Age (year)	68.34 ± 7.29	69.18 ± 8.09	0.645	0.520
Gender (male/female)	43/27	47/23	0.450	0.481
BMI (kg/m^2^)	24.34 ± 3.13	23.58 ± 2.65	1.550	0.123
ASA (II/III)	46/24	42/28	0.490	0.484
TNM stage			0.282	0.595
T1N0M0	63	61		
T2N0M0	7	9		
Position (left/right)	33/37	41/29		
Diameter (cm)	4.56 ± 1.03	4.37 ± 0.92	1.151	0.252

**Table 2:** Comparison of extubation time, breathing recovery time, and wake-up time between the two groups ($\bar{x}$ ± *s*, min).

	Extubation time	Respiratory recovery time	Awakening time
Control group (*n* = 70)	15.17 ± 3.22	12.39 ± 3.24	14.18 ± 4.19
Study group (*n* = 70)	16.26 ± 3.76	11.72 ± 3.81	15.41 ± 4.82
*t*	1.842	1.121	1.611
*P* value	0.068	0.264	0.109

**Table 3:** Comparison of postoperative pain between the two groups ($\bar{x}$ ± *s*, points).

	Awake	12 hours after awake	24 hours after awake
Control group (*n* = 70)	5.53 ± 1.03	1.83 ± 0.46	1.31 ± 0.32
Study group (*n* = 70)	5.01 ± 1.24	1.43 ± 0.38	1.27 ± 0.27
*t*	2.699	5.609	1.399
*P* value	0.008	<0.001	0.164

**Table 4:** Comparison of adverse reactions.

	Fever	Nausea and vomiting	Hypohemoglobin	Others	Total adverse reactions rate
Control group (*n* = 70)	2	1	1	2	6 (8.57%)
Study group (*n* = 70)	1	3	2	2	8 (11.43%)
*χ*2					0.318
*P* value					0.573

## Data Availability

The datasets used during the present study are available from the corresponding author upon reasonable request.
